# Supplementary figures and images for: Using cluster and rest redistribution set structures as alternatives to resistance training prescription method based on velocity loss thresholds
Source: PeerJ. 2022 Mar 29;10:e13195. doi: 10.7717/peerj.13195 (PMC8973460; doi:10.7717/peerj.13195)

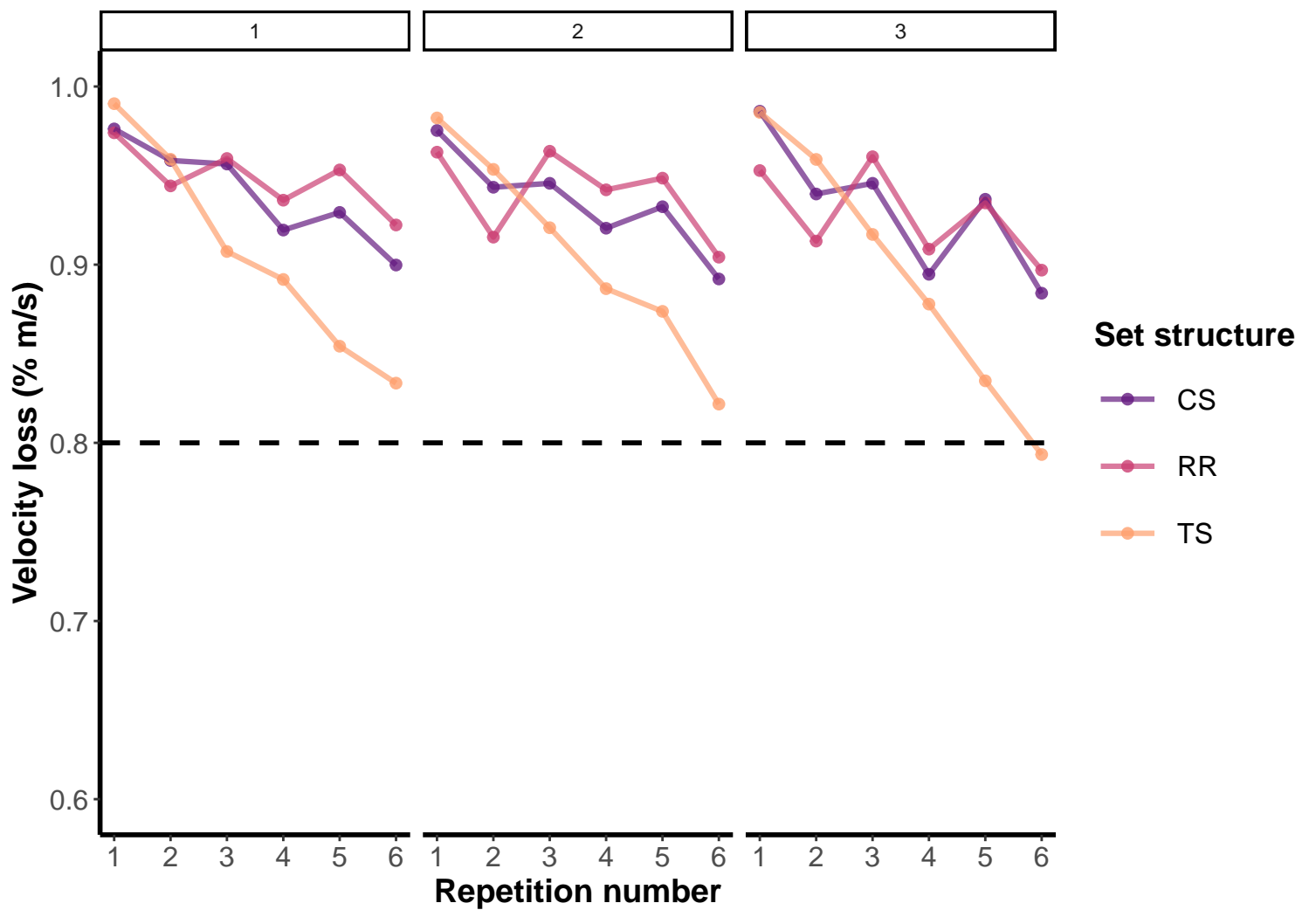

Supplement: Supplemental Information 1 — The dashed line represents a 20% velocity loss threshold whereas each dot represents average velocity of every repetition across three sets expressed as percentage of the fastest repetition. CS, Cluster set structure; RR, Rest redistribution set structure; SQ, Back squat; TS, Traditional set structure [file peerj-10-13195-s001.pdf]

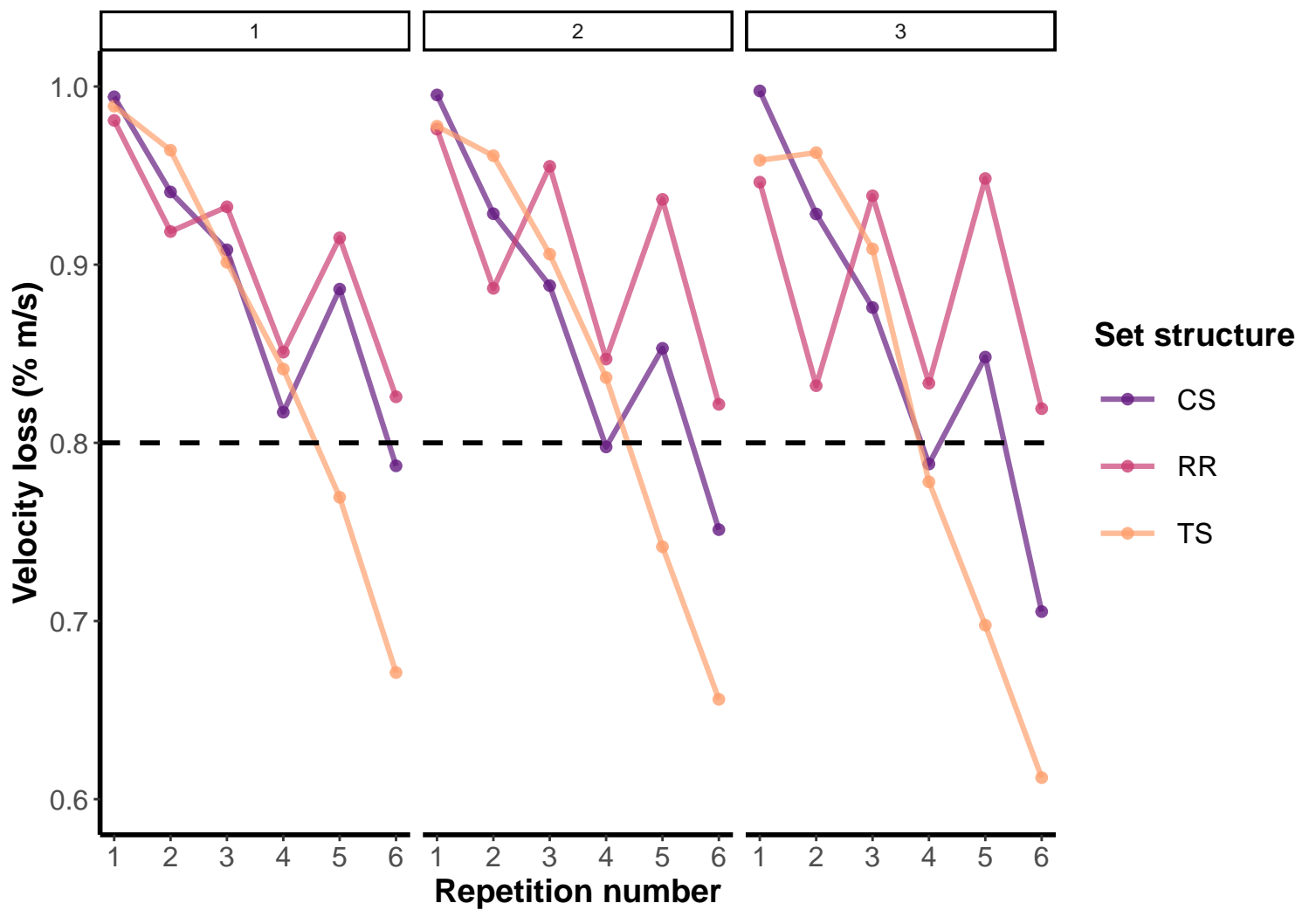

Supplement: Supplemental Information 2 — The dashed line represents a 20% velocity loss threshold whereas each dot represents average velocity of every repetition across three sets expressed as percentage of the fastest repetition. BP, Bench press; CS, Cluster set structure; RR, Rest redistribution set structure; TS, Traditional set structure [file peerj-10-13195-s002.pdf]
